# Advances in biomarkers and diagnostic significance of organ aging

**DOI:** 10.1016/j.fmre.2023.03.009

**Published:** 2023-04-09

**Authors:** Yulin Chen, Jiadong Li, Xinru Liu, Zhen Geng, Ke Xu, Jiacan Su

**Affiliations:** a Institute of Translational Medicine, Shanghai University, Shanghai 200444, China; b Organoid Research Center, Shanghai University, Shanghai 200444, China; c School of Medicine, Shanghai University, Shanghai 200444, China; d School of Life Sciences, Shanghai University, Shanghai 200444, China

**Keywords:** Aging, Biomarkers, Organ aging, Diagnose, Aging disease

## Abstract

A complete understanding of aging is a critical first step in treating age-related diseases and postponing aging dysfunction in the context of an aging global population. Aging in organisms is driven by related molecular alterations that gradually occur in many organs. There has previously been a wealth of knowledge of how cells behave as they age, but when aging is investigated as a disease, the discovery and selection of aging biomarkers and how to diagnose the aging of the organism are crucial. Here, we provide a summary of the state of the field and suggest future potential routes for research on organ senescence markers. We reviewed research on biomarkers of risk of aging from the perspective of organ aging and summarized the biomarkers currently used on three scales. We emphasize that the combination of traditional markers with emerging multifaceted biomarkers may be a better way to diagnose age-related diseases.

## 1 Introduction

With data showing that the global population has lived significantly longer since 1950, population aging may be one of the most important social trends of the 21st century [1]. By the midcentury, 16% of the global population is expected to be older than 65 years, which is the same proportion as the population under 12 years of age [2]. The proportion of people over 85 years is growing more than six times faster than the entire population [3]. The socioeconomic impact of the aging population is reflected in the increase in healthcare spending and the increased burden on social security and Medicare [4,5]. Inadequate financial and health security for the elderly leads to poor quality of life and susceptibility to disease in old age. One hundred eighty million of China's 264 million older people are affected by chronic diseases, that is, 3/4 of the older population, and the health resources required by the elderly are about 1.9 times the average consumption of the entire population [5]. This means the hospital management system must increase the proportion of departments for older people.

The World Health Organization (WHO) defines aging as a chronic and long-term process in which molecular and cellular damage in the organism gradually accumulates over time. This random accumulation of harmful events will eventually lead to health decline and a variety of chronic diseases, including cardiovascular and neurodegenerative diseases, bone-related diseases, and cancer [6]. The WHO also noted that the future direction of public health actions for healthy aging should be directed toward maintaining normal physiological functions rather than curing diseases [7]. Therefore, diagnosis and treatment at the early stage of the disease may help maintain normal physiological function. Several hallmarks of aging have been identified as key markers for the diagnosis of aging diseases, and the selection of biological markers for assessing organ aging status uses biological age to accurately assess the degree of aging in individuals [8,9]. In current studies, the accumulation of senescent cells has been shown to influence biological aging and lead to the emergence of age-related pathologies. Identifying senescence markers with high sensitivity and specificity for the characteristics of cellular senescence is the key to slowing the development of age-related diseases (ARD) [10,11]. Therefore, the combined application of aging markers with disease markers in diagnosing and treating diseases, that is, measuring individual aging by combining biological age and chronological age, may be an important future research direction for geriatric diseases. Here, we discuss organ aging markers at three levels and then summarize the aging markers identified in aging organs (including the cardiac, brain, skeleton, and liver) and the clinical use of aging marker assays. Constructing an index system to measure organ aging status for the diagnosis of aging-related diseases provides a reference and research direction for future exploration of aging markers to improve diagnostic efficiency, delay disease progression, and protect the health of the older population.

## 2 The three transversal elements of aging

**Fig. 1:**
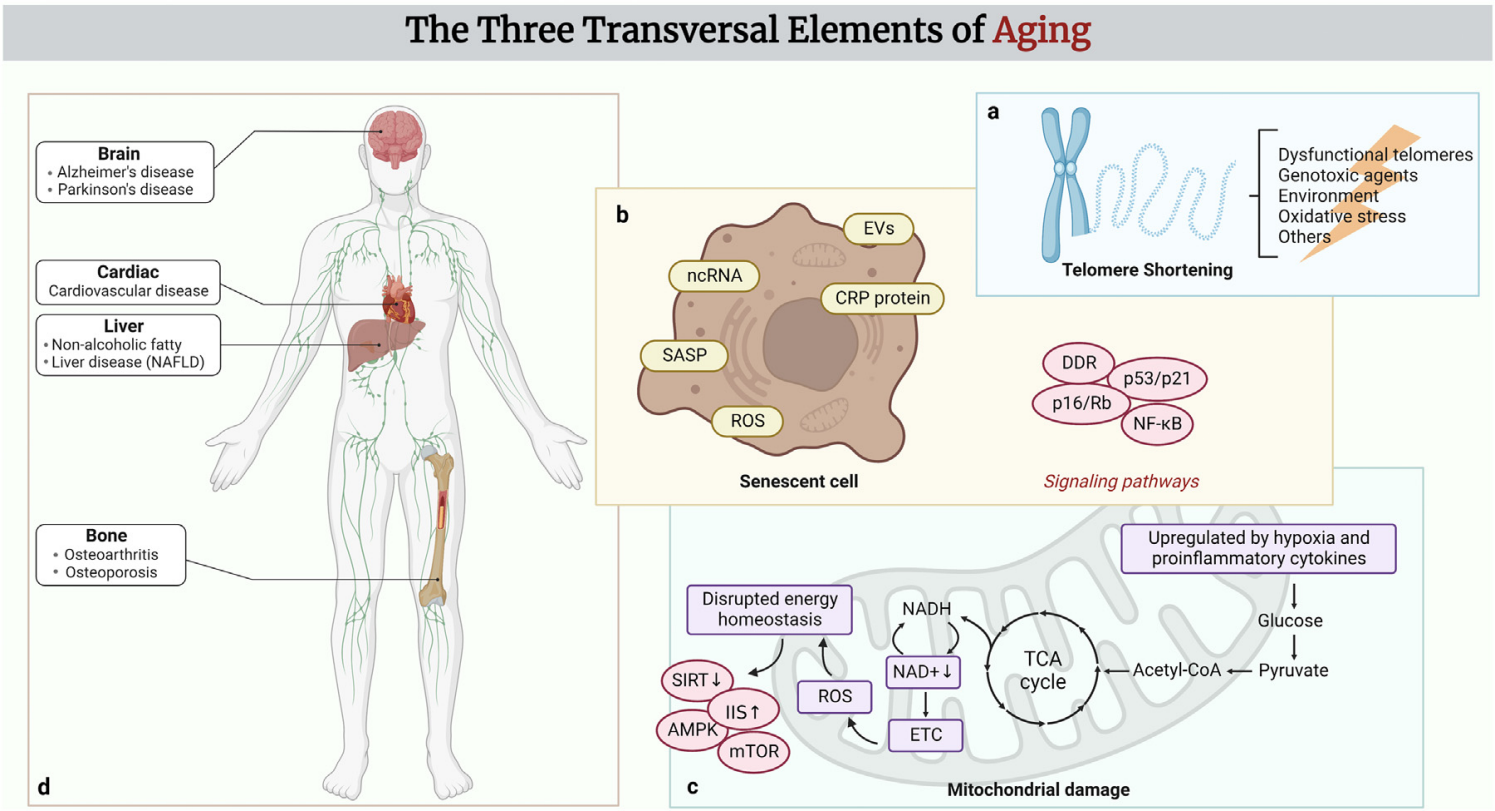
**The three transversal elements of aging**. (a) Stimulated by external signals, mammalian aging is accompanied by an accumulation of telomeric DNA damage responses and loss of telomerase, triggering telomere shortening and attrition. (b) Bioactive molecules that are abnormally secreted during cellular senescence may be excellent biomarkers of aging, which can be detected by technology in the blood. (c) Metabolic disorders and imbalance in metabolite secretion in senescent cells will consequently lead to aberrant aging signaling pathways and diseases. (d) Evidence of multiple aging mechanisms triggering organ aging. Created with BioRender.com.

Advancements in aging research have been made in recent years. In a review published in *Cell* in 2013, aging was divided into nine common characteristics that manifest as people age, aggravate these traits to accelerate aging, and interfere with them to slow down aging [12]. Although these features are interrelated, we assume that, to some extent, these features are hierarchical, and the following sections of this paper describe the markers of aging in greater detail at three levels (Fig. 1).

### 2.1 Telomere shortening and attrition

Telomeres are a complex sequence of noncoding bases located at the end of eukaryotic chromosomes. They comprise hundreds of repetitive DNA sequences TTAGGG and telomere-binding proteins called Shelterin (six telomeric proteins, including TRF1, TRF2, RAP1, TIN2, TPP1, and POT1), which do not carry genetic information [13]. Their main function is to prevent chromosome instability during DNA replication by capping the chromosome ends [14]. The telomere length shortens in normal adult somatic cells as cell replications increase, because DNA polymerase cannot act on the 3′ ends of single-stranded DNA [15]. At some point, telomeres lose their ability to cap DNA, and the uncapped state results in the loss of protection of the chromosome ends [15]. This causes a DNA damage repair (DDR) response, ultimately leading to cellular senescence and death [15]. Telomerase is crucial in this process to prevent telomere loss, and is the main cellular protein responsible for telomere maintenance and lengthening [16]. However, telomerase expression is strictly regulated, inactivated in most somatic and stem cells, and almost exclusively present in germ cells [17]. Specifically, multiple external stimulus signals activate DNA damage-binding proteins, and downstream signal transduction pathways are activated to transmit the signal to receptors [17]. Telomeric DNA damage signals are enhanced by kinase cascades, and effectors initiate regulation of their repair responses; DNA damage repair pathways are activated, and cell fate progresses toward apoptosis and/or senescence [18].

Although cell senescence does not necessarily imply aging of the organism, telomere shortening has been observed in both conditions, and shorter telomeres are commonly seen in various pathological states [19,20]. Therefore, telomere length shortening has been studied as a risk marker for aging, in crosstalk with the development of common age-related diseases (ARD). At the organism level, telomeres become progressively shorter with individual age, with older individuals having significantly shorter telomeres than younger individuals [21]. An inverse association between relative leukocyte telomere length (LTL) and frailty was observed in older Finnish persons [22]. The relative LTLs in this cohort were 1.40 ± 0.29 in the 61-year-old population, while in the 71-year-old population, the relative LTLs were 0.86 ± 0.30. It is easy to understand that the initial length of the telomere varies from person to person. The rate of telomere shortening is inextricably linked to the depletion of replication during aging. As mentioned before, for tissues that proliferate faster, such as skin, the small intestine, and bone marrow, the increased number of cell divisions causes the accumulation of telomere wear, while for tissues that proliferate slower, such as heart, brain, and liver, oxygen free radicals and other harmful substances generated by cellular life activities also induce damage to the telomere sequence, causing telomere shortening [23], [24], [25]. Eventually, the accumulation of senescent cells occurs in each tissue and involves surrounding healthy cells, causing organ aging [26].

There are many methods for telomere length detection. The early Southern blotting (SB) technique was mostly used; although this method cannot detect the actual length of individual telomeres and can only report the overall length of cells, researchers still regard the method as the "gold standard" for telomere measurement [21]. Later, quantitative fluorescent in situ hybridization (Q-FISH) and flow fluorescent in situ hybridization (Flow-FISH) were developed based on a similar application of the principle of hybridization of labeled telomeres with fluorescent probes [27]. In 2002, Richard Cawthon first proposed the measurement of relative mean telomere length by quantitative PCR (Q-PCR), a fast and easy method, and later described a novel monoplex and multiplex Q-PCR method (mmQPCR) based on previous studies, which further reduced cost and error [28,29]. Telomere length measurement is highly susceptible to bias depending on the method. For example, different measurements for the same sample may yield inconsistent results [30]. This is why the telomere theory is still being studied in longitudinal studies [30].

### 2.2 Blood biomarkers

Cellular senescence is a highly complex process that can be activated by different stimuli and produce various responses, including cellular hypertrophy, nuclear and epigenetic rearrangements, and metabolic changes [31]. The previously mentioned telomere dysfunction that reaches a critical length, after activating the DNA damage response that leads to a senescent phenotype, is followed by a high level of sustained secretion of bioactive molecules by senescent cells [32]. Long-term increases in blood concentration of these reactive molecules will accelerate tissue fibrosis or necrosis, alter associated activities, and accelerate the onset of many age-related disorders [32]. These active molecules can be detected in the blood using appropriate tools, including numerous nucleic acids, chemokines, cytokines, growth factors, proteins, and exosomes [33]. We reviewed the studies on the contribution of these bioactive molecules to age-related diseases and their detection methods (Table 1). A new way of thinking and a new foundation for diagnosing and treating ARDs could lead to the discovery of aging biomarkers in the blood and research on their correlation with aging-related disorders.

**Table 1:** **Blood biomarkers for aging diseases**.

Category	Biomarkers	Expression level changes with age	Diseases	Testing Technology	References
Nucleic acid	miR-31-5p	Decrease	OP	qRT-PCR	[40]
	miR-21a-5p, miR214-3p, miR-30a-5p, miR-30d-5p	Increase	OA	miRNA microarray	[176]
	miR-130a	Decrease	ischemic vascular diseases	Next generation sequencing	[42]
	circSLC8A1	Increase	PD	RNA-seq	[48]
	hsa_circ_0032131	Increase	OA	qRT-PCR	[177]
	hsa_circ_0124644	Increase	coronary artery disease	circRNA microarray technology	[178]
	lncRNA BACE1	Increase	AD	qRT-PCR	[55]
	Lnc-LFAR1	Increase	hepatic fibrosis.	lncRNA microarray	[179]
Protein	GDF15	Increase	cardiovascular disease	SOMAscan assay	[59]
	STC1	Increase	cardiovascular disease; pulmonary fibrosis; AD	DIA mass spectrometry workflow	[180]
	SERPIN	Increase	cardiovascular disease	DIA mass spectrometry workflow	[180]
	β2-microglobulin	Increase	Brain aging	Western blotting	[63]
	Cadherin13	Decrease	OP	Plasma proteomic profiling	[181]
	CRP	Decrease	AD	multiplexed immunoassay	[67]
SASP	Inflammatory cytokines	Increase	Common diseases	qRT-PCR; Western blotting; Elisa	[182]
	Matrix metalloproteinases	Increase	Common diseases	qRT-PCR; Western blotting; Elisa	[183]
	chemokines	Increase	Common diseases	qRT-PCR; Western blotting; Elisa	[184]
Exosome	miR-27b, miR-199a, miR-185	Increase	OA	qRT-PCR	[185]
	P-tau	Increase	AD	Elisa; electrochemiluminescence assays	[186]
	exosomal-miR30e and miR-92	Increase	Atherosclerosis	RNA-seq	[187]

#### 2.2.1 Nucleic acid

Age-related changes in cellular transcriptome levels are also integral to physiological changes in cells, tissues, and organ systems associated with the aging process in multicellular organisms [34]. Transcriptome-based RNA sequencing analysis of many types of aging cells and organs has recently found numerous types of RNA especially expressed in aging samples, due to the development of next-generation sequencing (NGS) and the expanding use of bioinformatics analysis [35]. Among them, noncoding RNA (ncRNAs) is a current research hotspot [36]. Here, we will provide a focused review of microRNAs, circular RNAs, and long non-coding RNA that may serve as markers.

MicroRNAs (miRNAs) are single-stranded, endogenous, noncoding small RNA, approximately 22 nucleotides in length, that regulate gene expression at the post-transcriptional level by binding to target mRNAs [37]. Compared to traditional serological markers, circulating miRNAs have various advantages, such as high sensitivity, high specificity, and good stability [38]. Therefore, common detection methods, such as qRT-PCR and sequencing analysis, are available for analyzing miRNA expression in tissues [39]. Recently, circulating miRNAs have been excellent diagnostic tools for age-related bone diseases. Heilmeier et al. selected 10 miRNAs for serum analysis by qRT-PCR in 169 postmenopausal female study participants, combined with bone mineral density (BMD) on CT scans, to assess the association of miRNAs with the development of fragility fractures in older postmenopausal women, and found that combining changes in the levels of three miRNAs with BMD significantly improved the accuracy of fracture diagnosis [40]. MiRNAs also play a regulatory role in vascular aging, and upregulation of aging and inflammation-related miRNAs leads to vascular endothelial cell damage [41]. Dhahri et al. revealed by next-generation sequencing and qRT-PCR analysis that miR-130a expression was significantly reduced in aged mouse aortic endothelial cells (EC), overexpression of miR-130a in senescent EC reduced cell senescence and promoted angiogenesis [42]. In summary, serum miRNA expression profiles can be used as novel serum-based indicators of aging that may be more sensitive and specific than currently available markers for early detection [43]. However, differential miRNA expression can also be influenced by different patient characteristics, therefore it needs to be defined in combination with other criteria for disease diagnosis [44].

Circular RNA (circRNA) is an endogenous noncoding RNA with a covalent closed-loop structure widely present in various eukaryotic cells. It performs biological functions by acting as a microRNA molecular sponge, interacting with RNA-binding proteins, regulating gene transcription, and encoding proteins [45]. CircRNAs are resistant to nucleic acid exonucleases and are not easily degraded because they do not possess polar structures at the 5′ and 3′ ends, thus it is characterized by more stable expression, high conservation and specificity [46]. Based on this stability, researchers have proposed the hypothesis of age accumulation of circRNA [47]. Hanan et al. found that circSLC8A1 was significantly up-regulated in the PD population by analyzing circRNAs comparing three regions in the brains of patients with PD and normal subjects, which may be involved in developing PD by sponging miR-128 to downregulate the expression of Sirt1 [48]. The age accumulation trend of circRNA implies its potential as a biomarker of aging, not only enriched in body fluids, but also specifically detected in free-floating cells in these fluids (e.g., circulating blood cells and circulating tumor cells), which supports circRNA as a biomarker of aging [49,50].

Long noncoding RNA (lncRNA) is a type of noncoding RNA with transcripts longer than 200 bp, which were previously considered transcriptome transcriptional “noise” because of their lack of protein-coding function [51]. Transcriptome sequencing of proliferating and senescent phenotypic fibroblasts revealed many differentially expressed lncRNAs in senescent cells [52]. It showed that lncRNAs regulate gene expression at multiple levels in RNAs, which can affect cell growth and aging, as well as developing age-related diseases, such as neurodegenerative and cardiovascular diseases, through multiple pathways [53,54]. In the plasma of Alzheimer's patients, lncRNA levels of BACE1 increased markedly compared to controls. This may drive disease progression by promoting amyloid Aβ production, while levels of other lncRNAs associated with AD did not change significantly [55]. lncRNAs are closely associated with the onset and development of many physiological and pathological processes. However, the relationship between lncRNAs and cellular senescence is still poorly studied. Currently, research on lncRNAs and diseases is still focused on neurological and cardiovascular diseases. With the continuous use of sequencing analysis in the future, lncRNAs will provide new clues for the diagnosis of aging-related diseases.

#### 2.2.2 Proteins

First, the most well-known senescence-associated β-galactosidase (SA-β-Gal), whose expression increases with aging, is a recognized biomarker for senescence detection and can be distinguished from senescent cells by staining with an artificial substrate, X-gal [56].

Improved research techniques have given us the ability to mine for more specific markers. Biomarkers of aging show differential expression, not only on the transcriptome but also on specific proteomes [57]. Given the current increasing demand for early diagnosis and prognostic assessment of aging diseases, we can identify protein markers of aging by performing proteomic analysis in different types of aging cells or tissues to improve the recognition of aging, which is a multivariate, multidimensional event and has become a more promising diagnostic strategy [58]. Tanaka et al. used the SOMAscan assay to measure 1301 proteins in a cohort of 240 individuals aged 22–93 years. Growth differentiation factor 15 (GDF15) stood out as a risk marker for cardiovascular disease related to aging with increasing expression levels with age [59].

β2-Microglobulin (β2-MG) is a low-molecular-weight secretory protein found in the urine of patients with renal insufficiency [60]. Plasma levels of β2-MG are dynamically associated with various diseases and have been extensively studied in renal, autoimmune, and inflammatory diseases [61,62]. Smith et al. found that β2-MG expression in mouse plasma is up-regulated with aging and negatively regulates cognitive and neuroregenerative functions in the adult hippocampus [63]. In the brain, β2-MG can regulate normal brain development and synaptic plasticity behavior independently of its typical immune function, and aged mice with knockout β2-MG exhibit superior cognitive abilities in wild type (WT) mice [63]. Therefore, β2-MG might be used as a marker for CNS-related diseases.

C-reactive protein (CRP) is an acute temporal phase plasma protein that increases during systemic inflammatory response; in clinical practice, CRP has the characteristics of being easily detectable and actionable, and is a biological indicator that helps in the clinical diagnosis of diseases [64]. Due to the up-regulation of serum inflammatory factor expression during aging, and its close association with the pathophysiological development of several aging diseases, CRP is mediated by IL-6; therefore, CRP is used as a risk predictor of aging injury [64,65]. The altered CRP expression is observed in various diseases, but changes in CRP levels vary between diseases. For example, in older people with atherosclerosis, CRP levels are elevated, reflecting the degree of atherosclerosis [66]. In Alzheimer's patients, CRP levels in the serum are decreased compared with those before treatment, presumably due to using drugs with anti-inflammatory effects [67].

#### 2.2.3 Senescence-associated secretory phenotype (SASP)

The sustained DDR in senescent cells not only activates senescence signaling pathways in the form of telomere length shortening, resulting in abnormal expression of nucleic acids and senescence-specific protein molecules, accompanied by the release of a unique bioactive secretion, called senescence-associated secretory phenotype (SASP), in an autocrine manner, leading to increased cellular senescence [68]. It also affects surrounding cells through paracrine secretion, affecting their normal growth and differentiation processes, leading to organ and even systemic aging [69]. Most SASPs are associated with inflammatory, immunomodulatory, and tissue morphological changes, such as changes in Interleukin-1β, IL-6 and IL-8, matrix metalloproteinase (MMP) 3, MMP13, MMP9, CCL5, CCL7, CXC chemokine receptor1, CXCL2, and CXCL15 [70]. Whether it is harmful or not depends on numerous factors, such as the site of release, the time of stimulation, and the cellular response [71]. The expression of IL-6, 8, and 15 increased in senescent endothelial cells. MMP was also highly expressed in senescent endothelial cells and vascular smooth muscle cells [72]. Secretion of these SASPs is accompanied by an inflammatory response in the vasculature that manifests as atherosclerotic pathological features such as arterial plaque formation, changes in vascular remodeling, and thrombogenesis [73]. Some beneficial senescent cells may contribute to tissue repair and regeneration by transiently secreting SASPs and regulating the action of immune cells [74]. This positive effect is observed mainly in parenchymal and progenitor cells [74]. For example, when a liver injury occurs, SASPs secreted by senescent hepatic stellate cells (HSCs) recruit immune cells to attack HSCs and hinder further lesions of HSCs from causing serious liver diseases [69]. In addition, using SASPs as therapeutic targets to kill senescent cells has been shown to be an effective treatment for aging diseases [75,76].

SASP is an important research marker for aging, not only because of its wide variety of types and disease coverage but also because of its ease of detection. In contrast to tissue samples, blood can be extracted to analyze the expression level of SASP, and the detection of SASP at the molecular level is now mature, commonly used, including quantitative reverse transcription–polymerase chain reaction (qRT-PCR), western blotting (WB), and enzyme-linked immunosorbent assay (ELISA), as well as sequencing methods including DNA microarray and RNA sequencing [77].

#### 2.2.4 Extracellular vesicle

The involvement of nucleic acids, proteins, and some cytokines in cellular senescence through autocrine secretion has been extensively studied, whereas extracellular vesicles, a mediator of intercellular information exchange, have received a lot of attention recently. Consequently, attention has been turned to extracellular vesicles and their secreted contents by senescent cells, whose role as biomarkers and regulators of the aging process has attracted great interest from researchers [78]. The EVs chamber contains various bioactive molecules, covering the above-mentioned blood aging markers, such as proteins, DNA, noncoding RNA, and lipids, which are released into the body fluids with the EVs and play a regulatory role in several biological processes, including cell value addition, differentiation, and aging [79]. Most existing studies on senescent cell-derived exosomes overlap with senescence-associated bioactive molecules and are not reviewed here (Table 1).

The key prerequisite for detecting EV cargo is its isolation. Because ultrafiltration or ultra-ionization extraction methods, which are currently used more frequently, will cause many losses, there is still a lack of extraction methods to maximize the yield of EVs [80]. The vesicles have a strong research potential as a non-invasive disease detection method, but their translational application may be limited by the fact that they are not easy to extract and isolate [81]. These vesicles have strong research potential as a noninvasive disease detection method, but their translational application may be limited by the fact that they are not easy to extract and isolate. The microfluidic technique is currently proposed by researchers as a new strategy for vesicle isolation that may overcome the above problems, with great advantages regarding extraction time and quantity [81]. In the future, we must conduct more research on isolation and detection techniques in addition to investigating how EVs respond to aging diseases as biomarkers.

### 2.3 Metabolic alterations

In 2019, the International Cell Senescence Association (ICSA) defined the key molecular characteristics of cellular senescence, of which metabolic disorders are an important feature [82]. Molecular changes within cells and intercellular interactions during aging lead to changes in the microenvironment and dysregulation of the metabolism of the main intracellular nutrients (glucose, lipids, and amino acids), resulting in disturbances in the metabolism of nicotinamide adenine dinucleotides (NAD^+^), further accelerating cell senescence [83]. In this section, we will discuss the metabolic regulation of senescent cells, how this is reflected in organ aging, and triggers age-related diseases. Understanding the deregulation of mitochondrial homeostasis during aging is important for preventing and treating disease.

Mitochondria play a key role in the most fundamental energy conversion processes in the cell, as a place for the final oxidation of glucose, lipids, and amino acids to release energy [84]. In normal conditions, glucose entering the cell through glycolysis produces the end product pyruvate, which will be metabolized to acetyl coenzyme A (acetyl-CoA), NAD^+^ is converted to its reduced form NADH [85]. Acetyl-CoA will then participate in the tricarboxylic acid (TCA) cycle in mitochondria, and most of the ATP required for cellular activity is produced by oxidative phosphorylation (OXPHOS) on the inner mitochondrial membrane [85]. In senescent cells, mitochondrial damage and reduced function lead to metabolic abnormalities, as shown by a decreased NAD^+^/NADH ratio, increased ROS levels, decreased ATP production, and increased AMP production [86]. The phenomenon in which the expression level of NAD^+^decreases with age has been observed in both mice and humans [87,88]. In AD model mouse brains, a reduced NAD^+^/NADH ratio and impaired metabolic function of brain energy were observed [89]. Pharmacological treatment with NAD^+^ supplementation restored neural progenitor cell viability, increased sirtuin levels, restored mitochondrial dysfunction to a certain degree, reduced neuroinflammation, and behavioral restoration of some cognitive abilities, suggesting a critical role in NAD^+^ metabolic levels in AD [89]. These and other data indicate that changes in mitochondrial energy metabolite levels are strongly associated with aging, and using metabolic indicators to aid the diagnosis of the disease may be a strategy to improve diagnostic efficiency. Current assay techniques for cellular energy metabolism are standardized, and kits are available to measure different indicators of mitochondrial function and assess mitochondrial function by the rate of cellular electron influx and passage through the electron transport chain [90].

The products of mitochondrial energy metabolism are involved in the signaling pathways that regulate aging. Reduced NAD^+^ expression inhibits the activity of NAD^+^ -dependent deacetylases (sirtuins). For example, in aged mouse endothelial cells, where SITR1 activity is reduced and angiogenesis is decreased, the up-regulation of NAD^+^ levels restores SITR1 activity and increases mitochondrial function [91]. The signaling molecule AMP-activated protein kinase (AMPK) is stimulated by changes in the AMP/ATP ratio, and the level of AMPK activation decreases as individuals’ age. Using AMPK activators can effectively inhibit aging; on the contrary, the knockdown of the AMPK gene causes elevated intracellular reactive oxygen species (ROS) levels and accelerates the aging process [92]. In conclusion, multiple aging-related signaling pathways regulate mitochondrial energy metabolism to maintain a dynamic and stable state of health that would otherwise lead to the outcomes of age-related metabolic diseases.

## 3 Heart

**Fig. 2:**
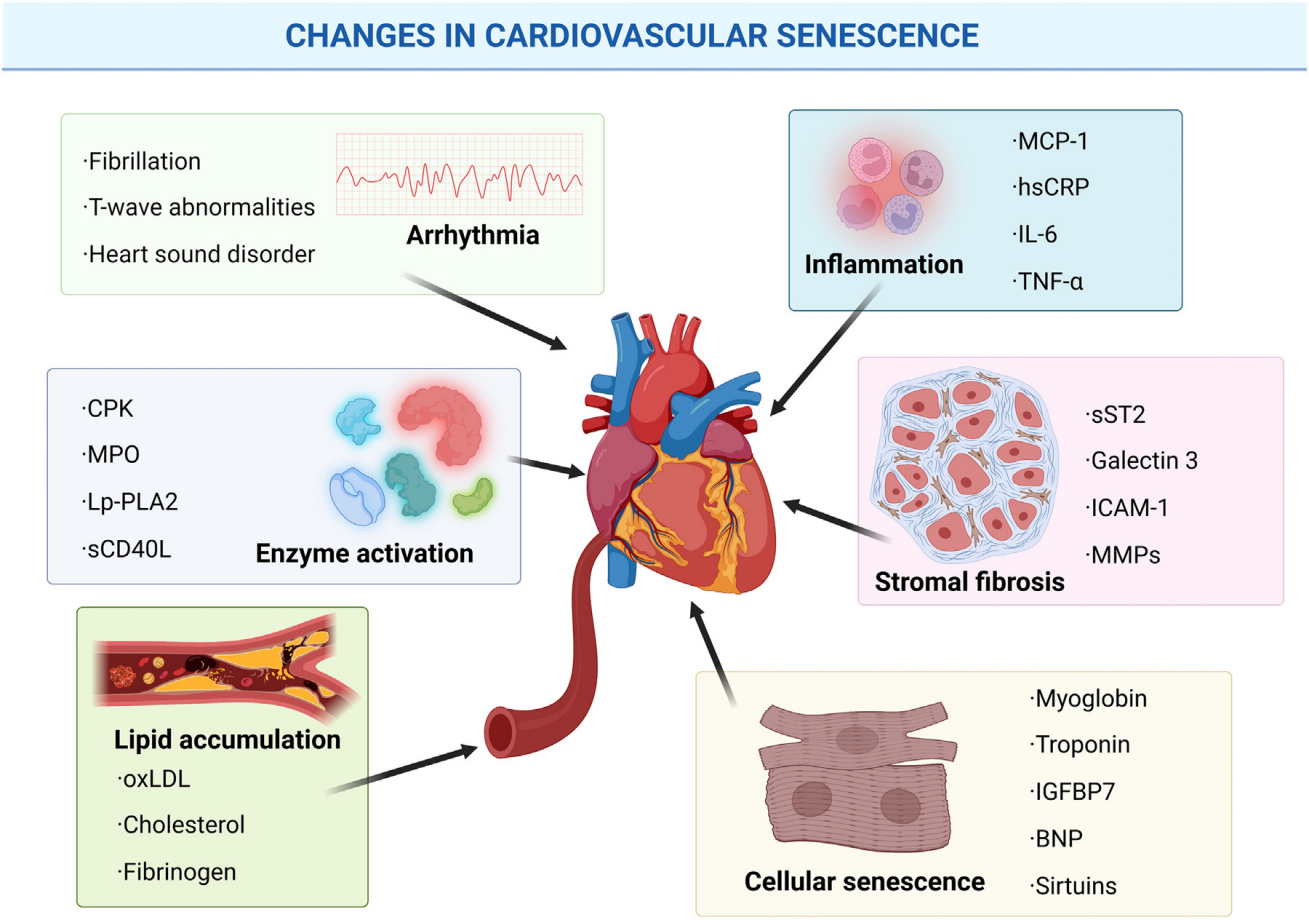
**Classification of cardiac aging markers according to pathophysiological processes**. In different cardiac senescence processes, the presence of characteristic biomarkers predicts the senescence of cardiomyocytes or fibroblasts. Created with BioRender.com.

Cardiovascular diseases (CVD) are a group of diseases involving the heart or blood vessels, including atherosclerosis, heart failure, and myocardial infarction. Individuals with CVD or those at high risk (due to one or more risk factors, such as hypertension or diabetes) need to be identified and managed with precise and personalized diagnostic modalities [93]. Previous studies have found that many markers of aging are associated with the development of cardiovascular disease and provide a basis for cardiovascular risk scores (Fig. 2).

### 3.1 Atherosclerosis

Atherosclerotic plaques have been shown to be associated with premature cellular senescence. It is characterized by reduced cell proliferation, irreversible growth arrest and apoptosis, elevated DNA damage, epigenetic modifications, and shortening of the telomere [94]. Studies in different populations have suggested that the length of the telomere predicts the production of subclinical atherosclerotic markers [95]. The shorter relative telomere length of leukocytes may predict higher oxidized low-density lipoprotein (oxLDL), triglycerides, and high-sensitivity C-reactive protein (hsCRP) in the body [96]. OxLDL is believed to be produced by senescent smooth muscle or endothelial cells [97]. Macrophages transform into foam cells after phagocytosis of oxLDL [98]. Foam cells accumulate to form lipid streaks and plaques [98]. In large prospective cohort studies, increased levels of circulating oxLDL have been associated with an unfavorable prognosis of atherosclerosis [99]. A higher oxLDL would lead to a more than 2-fold increase in the risk of atherosclerosis in a 15-year follow-up of people aged 45–84 years [100]. Furthermore, Matsuo et al. confirmed the correlation of oxLDL with plaque vulnerability and fibrous cap rupture using optical coherence tomography (OCT) [101]. hsCRP is the most studied biomarker of inflammatory atherosclerosis and appears in the entire process of atherosclerosis [102]. hsCRP is secreted into the peripheral blood by foam cells in vascular plaques [102]. Numerous prospective clinical and multi-ethnic studies have demonstrated that hsCRP is an independent predictor of atherosclerosis. A study of 388 patients showed that elevated plasma levels of hsCRP were positively associated with the severity of atherosclerosis [103]. hsCRP is not only a predictor of atherosclerosis, but also allows for further assessment of the risk of cardiovascular disease based on the Framingham risk score [104].

### 3.2 Heart failure

Heart senescence often manifests itself as cardiomyocyte damage or endothelial cell dysfunction [105]. Impaired mitochondrial function in senescent cells induces telomere shortening and oxidative stress. In clinical trials, leukocyte telomere length was significantly associated with clinical outcomes in patients with ischemic heart failure [25]. Similar findings were further validated in larger-scale data (*n* = 10 000 samples) [106]. The shortening of telomeres increases the risk of heart failure [106]. This suggests that telomeres are diagnostic senescence markers and are applicable in CVD. Following mitochondrial dysfunction, a decrease in sirtuin 3 in the vasculature predicts the development of vascular hypertrophy and inflammation [107]. Sirtuin 3 knockout mice showed a significant increase in aging-related markers (SA-β-gal and p21) in the aorta [107]. Simultaneously, telomerase reverse transcriptase (TERT) levels were reduced by half [107]. These data support sirtuins as potential cardiovascular senescence markers. Gevaert et al. reported that intercellular adhesion molecule 1 (ICAM-1) and p53 are co-localized in endothelial cells of senescent mice [108]. Targeting endothelial cell senescence may be a novel therapy for heart failure. Therefore, early diagnosis of cardiovascular diseases by targeting aging markers will be a future research direction.

## 4 Brain

**Fig. 3:**
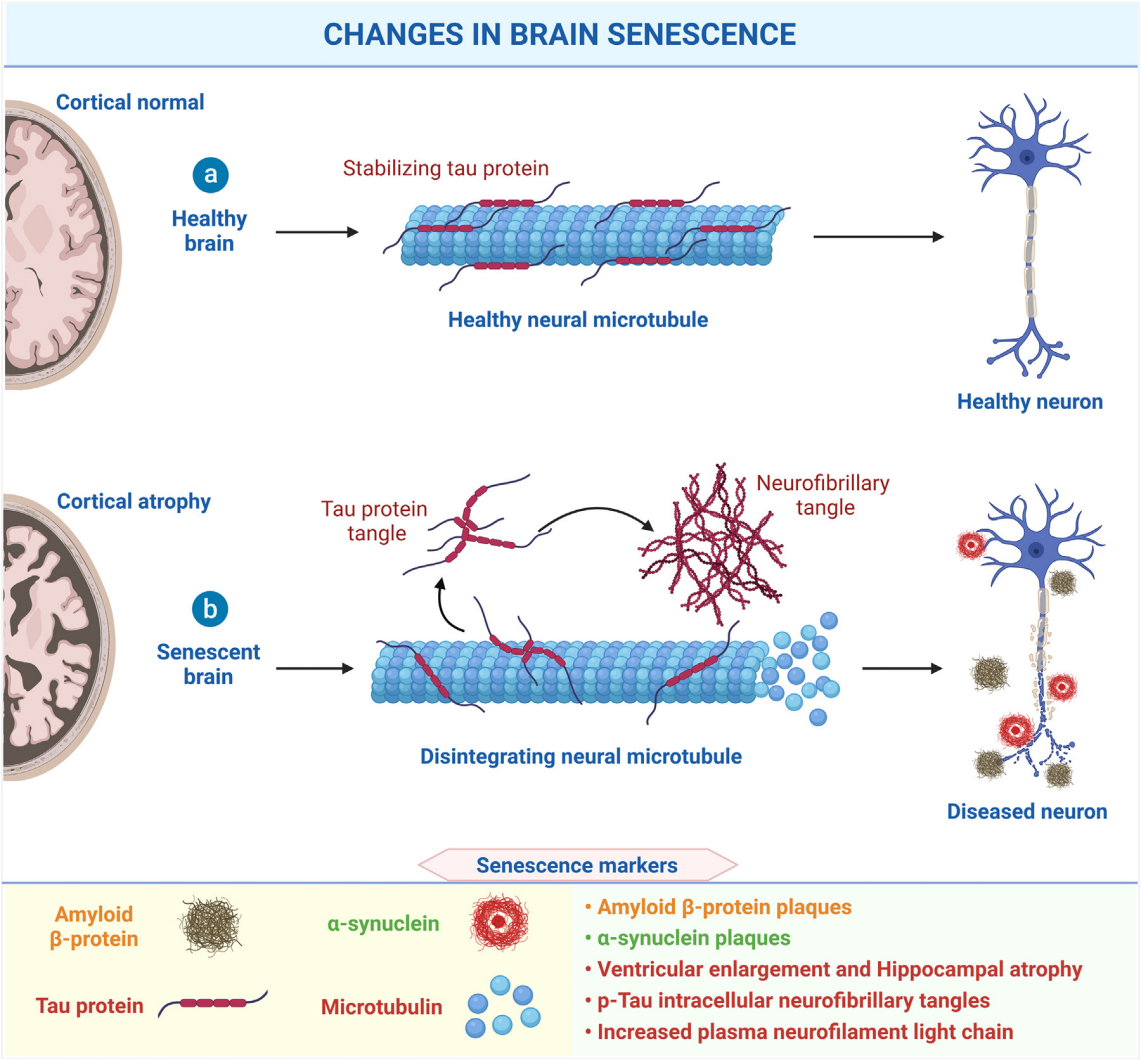
**Changes and biomarkers in brain senescence**. The characteristic pathological changes are cortical atrophy and a decrease in the number of memory neurons. During the aging process, Tau proteins and amyloid proteins form neural tangles deposited near the nerve fibers. Created with BioRender.com.

With age, brain senescence is inextricably linked to developing neurodegenerative diseases. Senescent neurons and glial cells severely impede the normal functioning of the brain [109]. Both neural tangles and amyloid accumulation lead to neuronal dysfunction and cortical atrophy (Fig. 3). The heterogeneity in brain senescence may lead to large differences in patient lifespan and the prevalence of neurodegenerative diseases (e.g., Alzheimer's disease and Parkinson's disease). Therefore, we need to assess and diagnose the progression of brain senescence from an early stage with reliable senescence markers.

### 4.1 Alzheimer's disease

Globally, Alzheimer's disease (AD) is the predominant neurodegenerative disease. Pathological features include amyloid β (Aβ)-containing plaque formation containing amyloid (A) and increased neurofibrillary tangles due to abnormal hyperphosphorylation of tau protein [110]. There are multiple oligomers of Aβ, of which Aβ42 is likely the culprit of cellular senescence [111]. Expression of the senescence marker p16 in primary neurons results in more Aβ42. Aβ42 accumulates in vascular endothelial cells and induces a senescent phenotype through the mediation of vascular endothelial growth factor receptor 1 (VEGFR-1), which poses a higher risk of cerebral amyloid angiopathy [111]. Similarly, tau protein has been shown to be a plasma marker of neuronal senescence. Tau is a microtubule-associated protein expressed mainly in brain cells, its role being to participate in microtubule polymerization and stabilize axonal microtubules [112]. Overactivated tau is associated with senescence of the brain's nervous system (astrocytes, microglia, and neurons) in patients with AD [113]. More p16-positive senescent cells and microglial fragments were observed near the enriched tau region [114]. Furthermore, the levels of Aβ and p-tau in the mouse brain cortex gradually increased when mitochondrial dysfunction occurred [115]. The above results confirm that different brain aging dimensions will eventually manifest as abnormal concentrations of Aβ and tau proteins.

### 4.2 Parkinson's disease

Parkinson's disease (PD) originates from the degeneration of dopamine neurons in the dense part of the substantia nigra of the mesencephalon [116]. Yoon et al. found that an excess of α-synuclein (α-syn) leads to DNA damage and cellular senescence in neuroblastoma cells [117]. α-syn treatment induces high p21 expression in astrocytes and microglia, which may be associated with mitochondrial dysfunction [117]. α-syn appears in the early stages of the disease and effectively distinguishes PD from other similar diseases. It is both cost-effective and reproducible, and can be used as a PD marker in clinical diagnostics. Concentrations of oligomeric α-syn and phosphorylated α-syn in cerebrospinal fluid (CSF) were positively correlated with the severity of PD, but their sensitivity and specificity remained unsatisfactory [118]. Due to the nonregenerative nature of neurons, the homeostasis of neuronal cells directly affects the process of brain senescence. Senescent astrocytes activate the p53/p21 pathway and release more glial fibrillary acid protein (GFAP) [119]. Therefore, abnormally high levels of CSF GFAP and serum GFAP based on ELISA are potential early markers of developing PD [120]. These different markers respond to various neurological senescence mechanisms, providing new insights into the future of PD diagnosis. Current research on brain senescence markers requires further refinement of assays and harmonizing prognostic models for closer clinical application.

## 5 Skeleton

**Fig. 4:**
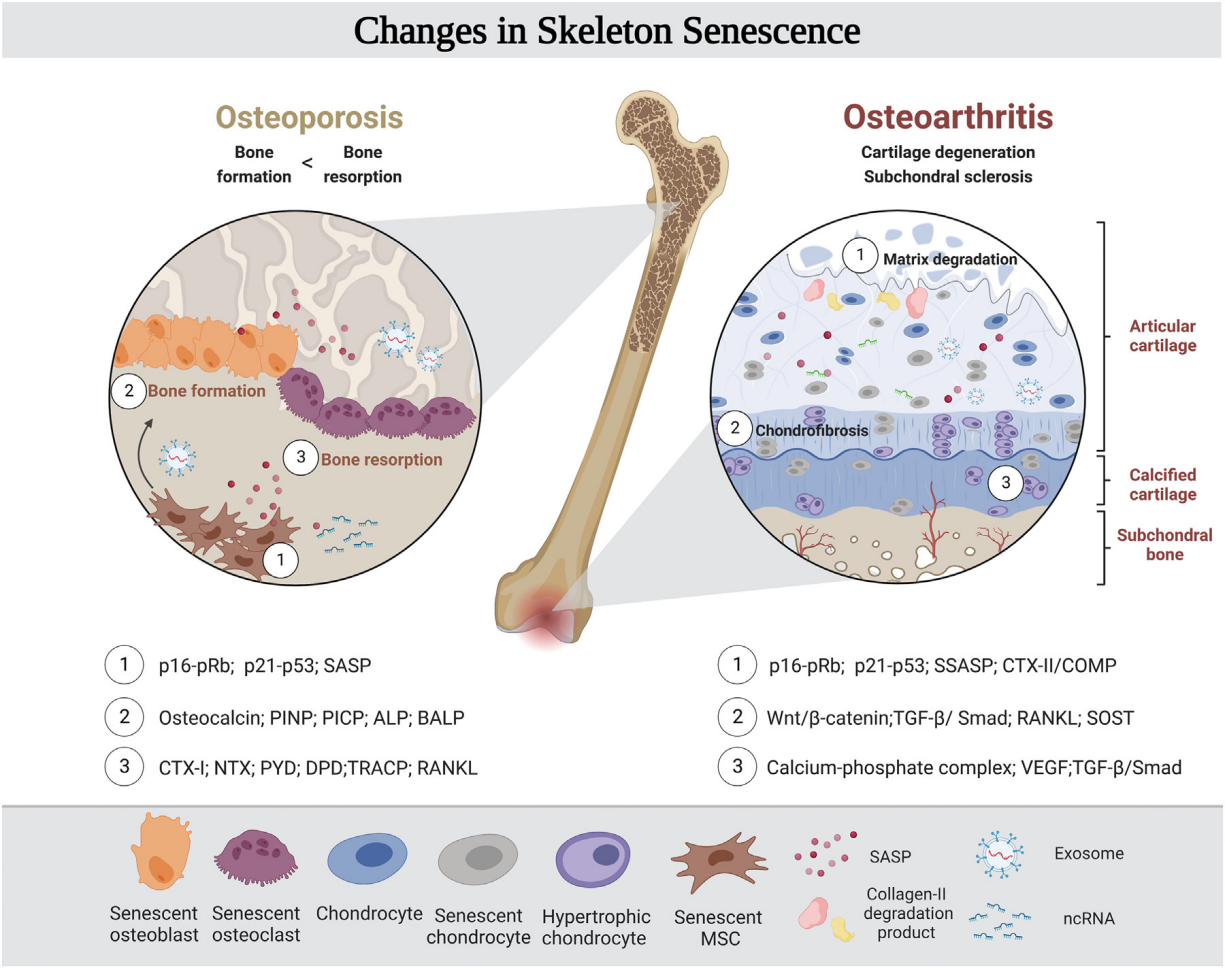
**Senescent biomarkers in the bone microenvironment**. The consequences of aging signals accumulating in the bone microenvironment is the loss of homeostasis of regulatory molecules and decline of skeletal organs' normal joint function, bringing out osteoarthritis and osteoporotic diseases. Catching the evolution pattern and biomarkers of organ aging and lesions in time will be conducive to the diagnosis and intervention of skeletal aging. Created with BioRender.com.

The skeleton is a dynamic organ precisely regulated by multiple mechanisms. As aging signals accumulate, aging-related changes in bone cells and related regulatory molecules will lead the organ to decay (Fig. 4). Changes in the physiological function of both cartilage and hard bones can cause diseases, including osteoarthritis and osteoporosis, sometimes accompanied by fractures, loss of normal joint function, and a series of other hazards that greatly affect the quality of life of older people in their old age [121]. The identification of biomarkers of bone aging will help identify high-risk patients and pioneer new and efficient treatments.

### 5.1 Osteoarthritis

The pathological features of osteoarthritis are characterized mainly by cartilage injury and subchondral bone remodeling [122]. Activation of DDR in senescent chondrocytes triggers changes in chondrocyte function. It is well known that in the microenvironment of the aging joint cavity, accumulation of DDR and high reactive oxygen levels stimulate chondrocyte senescence to secrete large amounts of SASP, including IL-1α, IL-6, IL-15, TNFα, and MMP13, which are highly expressed in the serum of patients with OA [123,124]. In particular, DDR accumulation also resulted in the telomere shortening observed in senescent chondrocytes. Southern blotting revealed that primary chondrocytes extracted from diseased joints of patients with OA had significantly shorter telomeres and higher SA-β-gal levels than normal chondrocytes [125].

An important role for chondrocytes in the joint is to provide type II collagen (COL-II), aggrecan, and other components for the synthesis and degradation of the extracellular matrix (ECM) [126]. With the accumulation of aging signals, collagen is gradually degraded under the action of MMPs. It has been proposed that the carboxy-terminal cross-linking telopeptide of type II collagen (CTX-II), a type II collagen degradation product, can be used as an OA marker, which is a class of markers that can be detected in the blood [127]. CTX-II concentrations in serum can be measured by ELISA or a fluoro-microbead guiding chip, reflecting the severity of OA [128]. In a GARP (Genetics, Arthrosis and Progression) study, radiological, pathological features of the knee, hip, hand, and vertebral joints in 302 subjects with OA were exceptionally well correlated with CTX-II levels [129]. Serum CTX-II levels in patients with knee osteoarthritis differed in different radiological grades, suggesting that combining both with radiological impact examinations can help diagnose the condition promptly and accurately at an early stage [129].

### 5.2 Osteoporosis

Osteoporosis (OP) is a classic age-related disease of bone metabolism characterized by low bone mineral density (BMD) and loss of bone mass and damage to microarchitecture, which are risk factors for fracture and increase the risk of fracture considerably [130]. Analysis of SASP in aged mouse cell populations revealed differential expression of SASP only in myeloid cells and bone cells, including CSF1-3, Hmgb1, Serpine1-2, and TNFα, and matrix metalloproteinases were significantly elevated compared to control young mice [131]. SASP is an excellent marker for identifying aging. Telomere shortening in bone mesenchymal stem cells exhibits senescent characteristics. In an osteoporosis model of mice deficient in telomerase, osteoblasts exhibit a senescent phenotype with markedly diminished differentiation and proliferation capacity, reduced bone formation, and increased bone mass loss [132]. Similarly, pathological manifestations of osteoporosis were observed in a mouse model that mimics a patient with telomere dysfunction [133]. A follow-up study of healthy older men whose telomere length was negatively correlated with age and telomere length showed a correlation with bone loss, possibly predicted by telomeres in OP [134].

Similar to cartilage degradation markers for OA, bone turnover markers (markers of bone resorption and bone formation, reflecting the number of osteoclasts) for OP can be used to predict the diagnosis [135]. The stable bone resorption marker commonly used in clinical practice is the cross-linked type I collagen telopeptide (CTX), and the bone formation marker is the N-terminal propeptide type I precollagen (PINP) [136]. Analysis of serum levels of CTX and PINP indicators, combined with BMD, age, and past fracture records, can initially evaluate the course of the disease and help avoid the risk of fracture [136].

## 6 Liver

**Fig. 5:**
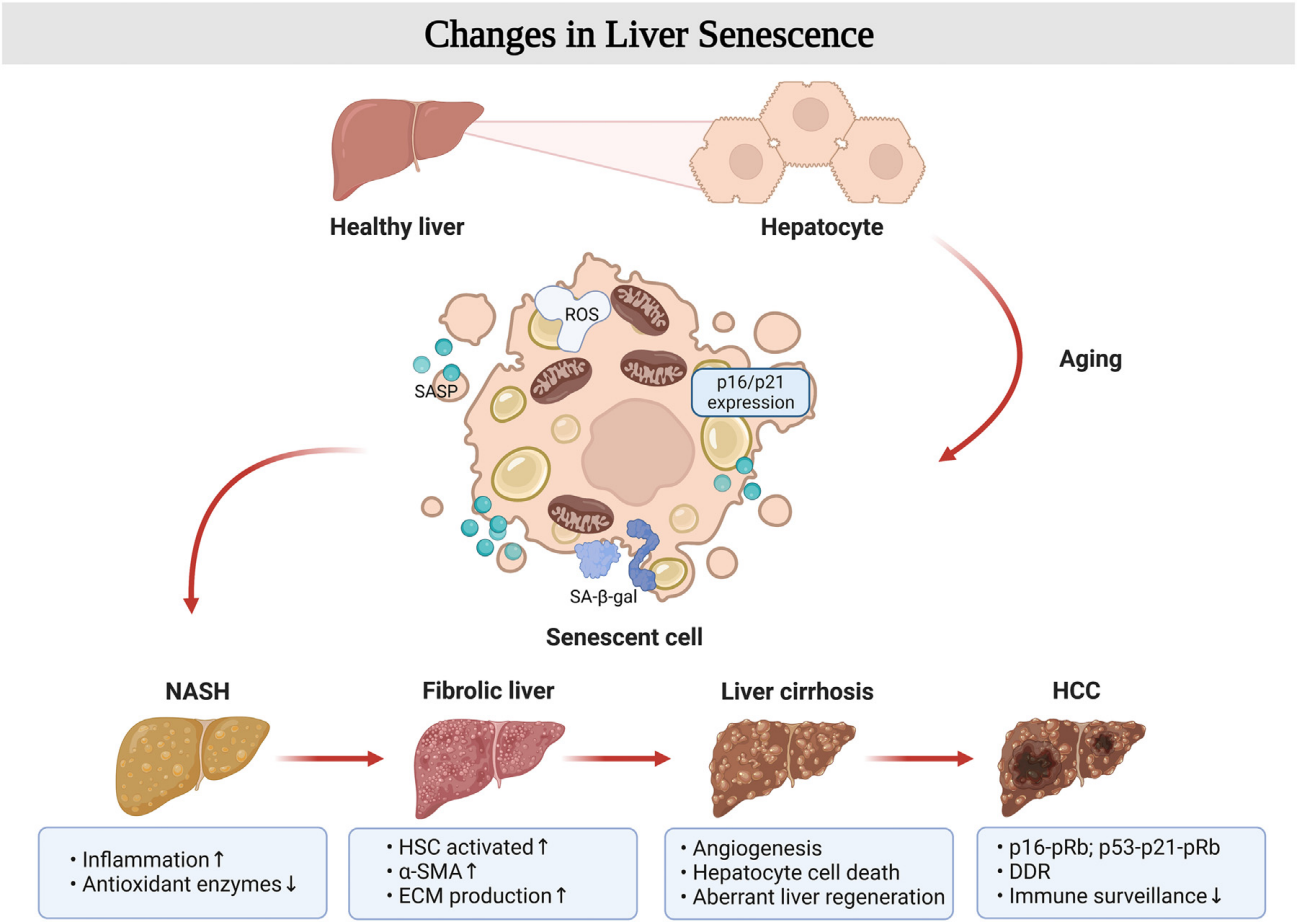
**The aging phenomenon in chronic liver disease**. With aging, fat accumulation and telomere shortening in hepatocytes, hepatic stellate cells (HSCs) in a resting state are activated by SASP and oxidative stress, which create an inflammatory environment and further promote telomere damage; HSCs secrete large amounts of α-SMA and collagen causing hepatic fibrosis; senescent liver sinusoidal endothelial cells (LSECs) have a reduced ability to regulate vascularization, leading to hepatic vascular dysfunction and impaired regeneration, and the liver develops toward cirrhosis in the aging process; the impaired function of senescent immune cells exacerbates the complexity of the intrahepatic environment, and the accumulation of these senescence markers in the liver creates the conditions for the induction of liver cancer. Created with BioRender.com.

Chronic liver disease (CLD) is more common in the elderly, but no liver disease occurs only in the elderly, so age may not be the etiology of liver disease. However, the effects of aging on the body and liver structure and function make the incidence, diagnosis, and management of all types of liver disease differ between the elderly and the young [137]. Studies have found that the incidence of non-alcoholic fatty liver disease (NAFLD) increases dramatically with age and has a worse prognosis. In the aged liver, senescent liver cells undergo all the cellular hallmarks of senescence-related changes that occur throughout the course of liver disease (Fig. 5). Aging is clearly a significant risk factor for developing CLD, and targeting both aging and disease characteristics in CLD diagnosis and treatment will help slow the development of CLD [138].

### 6.1 Non-alcoholic fatty liver disease (NAFLD)

Parenchymal liver cells make up 65% of liver cells and are the main functional operators of the liver. The number of hepatocytes gradually decreases, and their most important metabolic function diminishes as we age, leading to fat accumulation. In addition to this, inflammation, oxidative stress, and various age-related complications may trigger liver aging [139]. First, senescent hepatocytes have common features of cellular senescence, such as altered cell morphology, chromatin abnormalities, and cell cycle arrest [140]. A close association between hepatocyte senescence and NAFLD has been found, with DNA damage that accumulates in the liver of NAFLD patients, along with high expression of the classical senescence marker p21 in hepatocytes [140]. Telomere shortening was also observed in people with NAFLD, whose telomere length decreased at an average rate of 40 bp per year, much shorter than healthy controls of similar age [141]. Thus, hepatocyte senescence is an important feature of NAFLD, and its differential changes warrant further study as a marker for disease diagnosis.

Liver aging undergoes a shift from non-alcoholic steatohepatitis (NASH) to liver fibrosis, a process in which paracrine SASP that is secreted by senescent hepatocytes exacerbates aging [142]. Simultaneously, hepatic stellate cells (HSC) are stimulated to be activated from their resting state, specifically by enhanced synthesis of the extracellular matrix (ECM) and imbalance in the expression of enzymes responsible for the degradation of the ECM (matrix metalloproteinases, MMPs) and their inhibitors [143]. The abnormal accumulation of fibers in the liver causes liver fibrosis [143]. Up-regulation of the early liver fibrosis marker alpha-smooth muscle actin (α-SMA) was observed in a senescent hepatocyte model [144]. This phenomenon has also been studied in aged animal models, demonstrating that aging plays an important role in developing liver fibrosis [144].

Liver sinusoidal endothelial cells (LSECs), which account for the highest proportion of nonparenchymal cells in the liver, have a special open-window structure that makes these cells responsible for substance exchange and blood circulation in the liver, regulating the liver microenvironment, and maintaining normal metabolic functions of the liver, which are crucial players in the dynamic process of NASH and cirrhosis development [145]. Liver cirrhosis is one of the worsening outcomes of NAFLD, with a 5-year survival rate of only approximately 20% and an extremely high mortality rate [146]. It was shown that in aged rats with aging blood vessels, narrowing the LSECs window would cause pseudocapillarization of the structure, leading to dyslipidemia [147]. In clinical studies, although the expression of vascular endothelial growth factor (VEGF) in the liver of older patients with NAFLD did not change significantly, the level of sinusoidal perihepatic VEGFR2 was elevated, which may be associated with age-related pseudocapillarization [148]. Meanwhile, the liver microenvironment exhibited inflammation and oxidative stress, up-regulation of SASP expression, and decreased eNOS and p-eNOS proteins, showing differences between young and old rats [147]. When paracrine SASP secretion affects immune cells, it further aggravates the complexity of the liver microenvironment, and the accumulation of senescent cells in the liver creates conditions for inducting hepatocellular carcinoma [149].

Given the high risk of chronic liver disease, it is clinically significant for early diagnosis. The current diagnosis of NAFLD is based mainly on clinical and pathological characteristics, and liver biopsy result is the accepted gold standard [149]. However, this invasive test may cause errors due to sampling and does not allow dynamic observation, while the advantages of serum markers come into play. Therefore, the development of liver aging biomarkers with strong specificity and high sensitivity can provide value for diagnosis and improve the quality of treatment.

## 7 Others

In addition to the cardiac, brain, skeletal, and liver systems listed above, the endocrine system is related to the balance of the organism's internal environmental homeostasis, and its normal regulatory function may also be affected by degeneration and aging as one becomes older. This involves several endocrine organs, such as the pancreas, thyroid, and pineal gland, and may lead to circadian rhythm disorders, an increased incidence of diabetes, and an elevated risk of hypothyroidism [150].

### 7.1 Pancreas

The pancreas is an endocrine and exocrine organ simultaneously, which is essential to maintain energy metabolic balance and stable blood glucose levels [151]. Pancreatic aging is an important process in developing type 2 diabetes (T2D) [151]. It manifests itself as a weakening or loss of pancreatic β-cell function and decreased sensitivity to insulin and glucose levels [152]. With aging, senescent β-cells up-regulate Bcl-2 to resist apoptosis, leading to the accumulation of senescent cells in the islets [153]. In addition, it induces normal cell senescence through paracrine SASP. Single-cell sequencing revealed that aged mouse β-cells exhibited down-regulation of specific markers (e.g., PDX1 and Nkx6.1) and up-regulation of senescence markers (e.g., LDHA and p16^INK4a^) [154]. Similarly, an increase in the expression of the senescence markers CDKN1A, CDKN2A, and SASP was found in senescent α and β cells of aged crab-eating monkeys and human samples, implying that aged mammalian islets are enriched in senescent cells [155,156]. In another study, researchers identified IGF1R as a new marker of islet β-cell senescence [157]. This heterogeneous elevation of IGF1R is associated with an increase in p16^INK4a^, p53^BP1^, and aging-associated β-galactosidase, which increases significantly in response to metabolic stress and aging. Similarly, Aguayo et al. deleted the expression of p16^INK4a^ expression in INK-ATTAC transgenic mice, thus improving the function of senescent β-cells and reducing the SASP index [154]. Evidence suggests that with up-regulation of p21^Cis1^ in the early senescence phase, followed by p16 ^INK4a^, senescent islet cells are still in a reversible stress senescence process [154]. Once the advanced phase of SASP secretion is reached, cell senescence is irreversible. This implies that focusing on the detection of early aging markers is a more promising approach.

### 7.2 Thyroid

The thyroid is an important gland in the body and is essential for regulating metabolism. The level of thyroid stimulating hormone (TSH) secreted into blood serum is an indicator of thyroid function [158]. However, in several cross-sectional and longitudinal studies of aging, researchers have found that the upper limit of the TSH reference range varies by age, especially in older adults over 70 years of age, showing a significant increase in TSH levels. Furthermore, the increase in TSH levels may also be closely related to sex, geographical characteristics, physical conditions, and other indicators [159]. Older people with primary hypothyroidism and abnormal levels of TSH or FT4 may be more susceptible to atrial fibrillation and atherosclerosis [160]. Clinical diagnostic criteria for primary hyperthyroidism are abnormal levels of TSH and FT4, rather than hypothyroidism [160]. Clinical diagnosis is challenging to some extent due to gender differences or iodine intake and noncharacteristic clinical manifestations, which are very different in older patients than in younger people and are often confused with other characteristics of disease characteristics of older people, to the detriment of disease diagnosis and treatment [161].

### 7.3 Pineal gland

The pineal gland, located in the central region of the brain, is the most essential endocrine gland that regulates the circadian rhythm [162]. As the pineal gland ages, the most striking features are the calcification of the pineal gland and reduced melatonin secretion [163]. Several reports have noted a strong association between maximum pineal gland calcification and several neurodegenerative diseases, including AD [163]. Most interestingly, X-ray phase-contrast tomography showed significant degenerative changes in the calcified pineal gland of patients with AD, that is, abnormal delamination, fissures, and hollows in the calcified areas [163]. The incidence of pineal gland calcification in adults over 30 years old reached 83%, while this rate in high-aged turkeys and rats would reach 100% [164]. This implies that pineal gland calcification is universal and synchronized with individual aging. Furthermore, melatonin acts as an endogenous antioxidant; removing the pineal gland or inhibiting melatonin accelerates the aging process in rodents. Compared to younger people, older people had substantially lower levels of melatonin metabolites in their urine, without significant differences in circadian rhythm. When the pineal gland senesces, low levels of melatonin are unable to mitigate the neurological injury caused by ROS, which in turn exacerbates the risk of brain disease [165]. Melatonin levels in AD patients without any neuro plaque or neurofibrillary tangle have been reported to decrease from 280 pg/mL to 39 pg/mL in the CSF [166]. This finding indicated that altered melatonin secretion might be an early event in neurological senescence, even before the onset of clinical symptoms. From the above evidence, it appears that calcification-induced pineal gland endocrine degeneration is directly associated with senescence. Focusing on the calcification of the pineal gland and melatonin release may be a promising indicator of aging.

## 8 Application of biomarkers of aging

In aging research, aging biomarkers can be used not only to diagnose diseases but also as therapeutic targets. A landmark study in 2015 reported that senolytics, a class of drug combinations that selectively kill senescent cells, consisting of two drugs that selectively kill senescent cells, dasatinib and quercetin, exhibited a delayed effect on the pathological features of aging in several organs after treatment in aged mice, with a certain anti-aging therapeutic effect [167]. Subsequently, this drug combination was tested in clinical trials for the first time in 2019, in older adults with idiopathic pulmonary fibrosis (IPF), who were treated for three weeks and showed improvements in basic physiological functions such as walking distance and speed, sitting and standing, but without measurable effect on disease-related cardiorespiratory failure, suggesting that future senolytic drugs may require a long period of study [168].

Fluctuations in NAD^+^ levels during aging greatly affect the metabolism of elderly people and can be increased by supplementation with NAD^+^ precursors such as nicotinamide (NAM) [169]. In 2018, Mitchell et al. reported that NAM improved lifespan in mice chronically high-fat diet (HFD), prolonged survival days with NAM supplementation, improved hepatic fat accumulation, and reduced morphological alterations, along with restored glucose catabolic function of the liver and increased sirtuin activity [170]. The feasibility of this strategy has also been demonstrated in clinical studies, where the long-term administration of nicotinamide riboside chloride (NIAGEN®) in middle-aged and older adults effectively improved NAD^+^ metabolism, which not only maintains cardiovascular health, but may also have beneficial effects on metabolic diseases such as diabetes, and no significant toxic side effects were observed [171].

In addition, using aging markers as components of adaptive materials for disease treatment in translational medicine research is a highly interesting research direction. In bone disease research, because MMP13 is highly expressed in the joint microenvironment of patients with osteoarthritis, Nagase et al. developed a peptide fluorescent probe with strong selectivity for MMP13 that will sever the connection between the response element and the carrier in an environment of high concentrations of MMP13, emitting intense fluorescence, which can clearly distinguish healthy joints from OA joints 8 weeks after destabilization of the medial meniscus (DMM) and is an effective tool for monitoring in the early stages of OA [172].

## 9 Conclusion and future perspectives

Medical issues among older people have caught the interest of experts as population aging has emerged as a major global trend. Timely assessment and diagnosis in the early stages of aging not only improve the patient's prognosis but also reduce social, medical, and economic burdens, which have far-reaching clinical and practical significance. The mechanism underlying the aging process in humans is incredibly complicated. Despite numerous studies showing a link between different biomarkers and the onset of aging, current aging markers are not without flaws. For example, there is a lack of highly sensitive and specific biomarkers that can be used to routinely test for aging, as well as a lack of aging markers that can accurately measure aging. As a result, it is difficult to conduct preventive measures against aging and the diseases associated with it without indicators to measure individual aging. Therefore, a complete search for suitable aging biomarkers using a multi-omics approach can offer new perspectives and a foundation for identifying and managing aging-related disorders.

In this review, we categorized the nine main senescence indicators into three categories and listed the currently achievable detection techniques. Telomere length changes include aspects of genomic instability and epigenetic changes, and as this damage builds up over time in the cell, cellular senescence results. Blood contains some biomarkers of aging, including proteins with defective structures caused by loss of proteostasis and bioactive molecules in the cellular aging microenvironment, while some aging-related intercellular communication signals are activated and can also serve as cues to capture aging. Furthermore, an imbalance in the metabolism of aging organisms trigger changes in catabolic levels of nutrients in the body, which can be used as an important indicator to assess aging.

The dynamic equilibrium of aging tissues and organs is negatively impacted by interconnected intrinsic variables, and one aging disease frequently has a number of consequences, leading to many aging-related disorders in the elderly. Patients with NAFLD overlap highly with patients with type 2 diabetes and have similar markers of aging in their serum; both diseases are also associated with an increased prevalence of cardiovascular disease [173]. Additionally, when liver function deteriorates with age, the body's capacity to retain vitamin D, a critical component that supports bone health, also decreases, placing older people at increased risk of osteoporosis and fractures [174]. Not only does aging cause the pineal gland to calcify, shrink, and emit less melatonin, but these characteristics have also been observed in people with AD and are crucial clues for the diagnosis of AD [175]. Therefore, focusing on specific markers of organ aging would not only help diagnose associated diseases when marker levels change abnormally, but it may also signal the complications of other aging disorders.

All of this information highlights the need to examine the factors that contribute to human aging and to mine certain aging biomarkers to create a network of early molecular mechanisms of disease development with disease markers. Future aging assessment research will focus on combining disease characteristics with aging markers, using more practical detection techniques for timely diagnosis at the early stage of the disease, and providing the possibility of early detection and remission of age-related diseases. This will provide a better theoretical foundation and practical application value for improving human survival quality.

## Declaration of competing interest

The authors declare that they have no conflicts of interest in this work.
